# Phylogenetic detection of numerous gene duplications shared by animals, fungi and plants

**DOI:** 10.1186/gb-2010-11-4-r38

**Published:** 2010-04-06

**Authors:** Xiaofan Zhou, Zhenguo Lin, Hong Ma

**Affiliations:** 1 Department of Biology, the Pennsylvania State University, University Park, Pennsylvania 16802, USA; 2 Institute of Molecular Evolutionary Genetics, the Pennsylvania State University, University Park, Pennsylvania 16802, USA; 3 Intercollege Graduate Program in Cell and Developmental Biology, Huck Institutes of the Life Sciences, the Pennsylvania State University, University Park, Pennsylvania 16802, USA; 4 State Key Laboratory of Genetic Engineering, School of Life Sciences, Fudan University, Handan Road, Shanghai 200433, PR China; 5 Institute of Plant Biology, Fudan University, Handan Road, Shanghai 200433, PR China; 6 Center for Evolutionary Biology, School of Life Sciences, Fudan University, Handan Road, Shanghai 200433, PR China; 7 Institutes of Biomedical Sciences, Fudan University, Yixueyuan Road, Shanghai 200032, PR China; 8 Current address: Department of Ecology and Evolution, University of Chicago, 1101 E. 57th Street, Chicago, Illinois 60637, USA

## Abstract

**Background:**

Gene duplication is considered a major driving force for evolution of genetic novelty, thereby facilitating functional divergence and organismal diversity, including the process of speciation. Animals, fungi and plants are major eukaryotic kingdoms and the divergences between them are some of the most significant evolutionary events. Although gene duplications in each lineage have been studied extensively in various contexts, the extent of gene duplication prior to the split of plants and animals/fungi is not clear.

**Results:**

Here, we have studied gene duplications in early eukaryotes by phylogenetic relative dating. We have reconstructed gene families (with one or more orthogroups) with members from both animals/fungi and plants by using two different clustering strategies. Extensive phylogenetic analyses of the gene families show that, among nearly 2,600 orthogroups identified, at least 300 of them still retain duplication that occurred before the divergence of the three kingdoms. We further found evidence that such duplications were also detected in some highly divergent protists, suggesting that these duplication events occurred in the ancestors of most major extant eukaryotic groups.

**Conclusions:**

Our phylogenetic analyses show that numerous gene duplications happened at the early stage of eukaryotic evolution, probably before the separation of known major eukaryotic lineages. We discuss the implication of our results in the contexts of different models of eukaryotic phylogeny. One possible explanation for the large number of gene duplication events is one or more large-scale duplications, possibly whole genome or segmental duplication(s), which provides a genomic basis for the successful radiation of early eukaryotes.

## Abstract 2

A phylogentic analysis reveals that many gene duplications occurred prior to the split in animals, fungi and plants.

## Background

The history of eukaryotic evolution is one of ever-increasing diversity and complexity at multiple levels. The increases in genotypic and phenotypic complexity are usually associated with expansion of gene families. For instance, it has been shown that the diversification of gene families involved in cell differentiation and cell-cell communication contributed to the origination of multicellularity [1]. Other well-known examples are the MADS-box genes in plants [2] and olfactory receptor genes in animals [3]. These multigene families are subject to birth-and-death evolution and most new genes arise by gene duplication [3].

Gene duplication has been a ubiquitous phenomenon during eukaryotic history and has contributed to evolutionary innovation by generating additional genetic material for functional divergence and novelty [4]. After gene duplication, one of the duplicates might be released from selective pressure and have the potential to evolve new functions ('neofunctionalization') [4]. Alternatively, the two duplicates can accumulate different degenerative mutations and each retains a subset of the ancestral functions ('subfunctionalization') [5]. In addition, in certain situations, such subfunctionalization can lead to the optimization of subdivided ancestral functions in each duplicate, thus contributing to adaptation [6]. Besides its important role in the evolution of new gene functions, gene duplication also greatly contributes to the speciation process through the divergent resolution of duplicated genes in different populations [7]. Large-scale gene duplication events have been documented in animals and fungi, and are particularly frequent in plants [8-14] and are believed to be associated with dramatic increases in species diversity, such as the radiation of vertebrates and the diversification of flowering plants [15,16].

**Figure 1:**
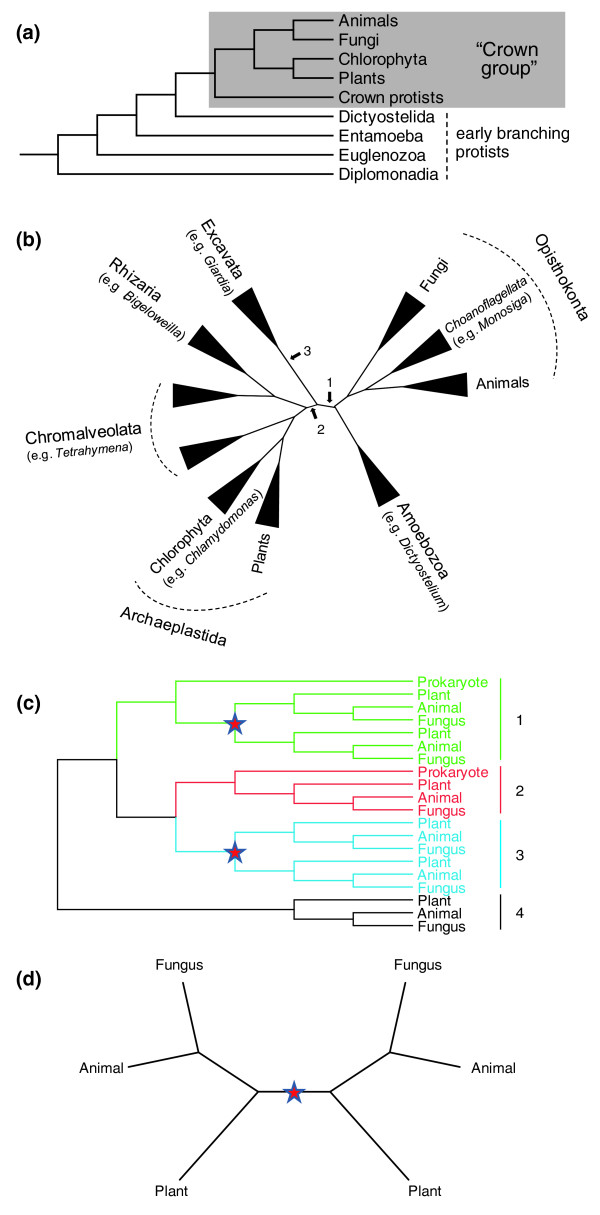
**Alternative views of the eukaryotic phylogeny and the design of phylogentic analysis**. **(a) **The 'crown-stem' topology of eukaryotic phylogeny. The topology shown is adopted from Sogin [18] and Sogin and Silberman [20]. **(b) **The 'six supergroups' classification of eukaryotes; the topology shown was reported by Hampl *et al*. [24]. Different hypotheses about the root position of the eukaryotic tree are indicated by numbered arrows: 1, the unikont-bikont hypothesis [26,27]; 2, the photosynthetic-nonphotosynthetic scenario [29]; 3, Excavata as basal group [28]. The branch lengths are arbitrary. **(c)** Hypothetical phylogenetic tree showing the definition of orthogroups in analyses I and III (see Results). Four possible orthogroup topologies are highlighted by colors: 1 (green), eukaryotic genes with prokaryotic outgroup and early eukaryotic duplication; 2 (red), eukaryotic genes with prokaryotic outgroup but no early eukaryotic duplication; 3 (blue), eukaryotic genes without prokaryotic outgroup but show early eukaryotic duplication; 4 (black), eukaryotic genes without prokaryotic outgroup nor early eukaryotic duplication. **(d)** Hypothetical phylogenetic tree showing an example of a eukaryote-specific gene cluster with duplication. The stars indicate gene duplications.

One of the most important evolutionary milestones is the early diversification of eukaryotes [17]. In the early 1990s, the 'crown-stem' model (Figure 1a) of eukaryotic phylogeny was proposed based on the study of small-subunit ribosomal RNA sequences [18-20]. This 'crown-stem' model suggests that plants, animals and fungi form a crown group in the eukaryotic tree and separated from each other more recently than some early branching protists. More recently, an alternative view of the early evolution of eukaryotes has emerged from phylogenomic studies and is increasingly accepted [21]. According to this view, eukaryotes are classified into six supergroups (Figure 1b): Archaeplastida (includes plants and green algae), Opisthokonta (includes animals and fungi) and four other supergroups of protists, including Excavata, a group of ancient protists that includes members with complex flagella and without functional mitochondria [21-23]. More recent studies further suggest that the number of supergroups might be more than six [24,25]. These supergroups would have diverged during the early phase of eukaryotic evolution, sometimes described as a 'Big Bang' event [17], although the diverging order of these supergroups is difficult to resolve and different root positions of the eukaryotic tree have been proposed [26-29]. In a number of scenarios, the split between Archaeplastida and Opisthokonta is among the earliest known eukaryotic divergences, before the divergence of other major protist groups from either Archaeplastida or Opisthokonta [26,27,29]. Therefore, the separation of plants from animals/fungi would be much more ancient than what was suggested by the 'crown-stem' model [18-20]. Even if the position of the root of the eukaryotic tree is between Excavata and the other supergroups, the split of the lineage with plants and the lineage with animals/fungi was still before those of several other protist groups, including Chromalveolata and Amoebozoa.

Previous phylogenetic studies of individual eukaryotic gene families for transcription regulators, kinesins, and recombinational proteins all indicate that there were duplication events before the split of animals and plants, suggestive of abundant gene duplication during early eukaryotic evolution [30-35]. This notion is also supported by a comparative genomic study, in which the established COG (prokaryotic clusters of orthologous groups) and KOG (eukaryotic clusters of orthologous groups) databases were used to reconstruct gene clusters and to analyze their phylogenies [36]. It was found that the inferred number of genes in the last eukaryotic common ancestor is 1.92-fold higher than in the first eukaryotic common ancestor, leading to the conclusion that early eukaryotes had significantly more gene duplication than prokaryotes during similar periods [36]. However, a systematic investigation of the extent of gene duplication prior to the split of plants and animals/fungi is still lacking. Here, we present extensive phylogenetic analyses of gene families and our results supporting the hypothesis that many of these families had experienced at least one duplication event before the divergence of the three major eukaryotic kingdoms.

## Results

### Reconstruction of gene clusters with the Markov Clustering Algorithm method

To identify gene duplication in early eukaryotic evolution, we reconstructed gene families from representative eukaryotic and prokaryotic species. The three multicellular eukaryotic kingdoms, plants, animals and fungi, belong to two of the six major eukaryotic supergroups (plants in Archaeplastida; animals and fungi both in Opisthokonta) [21]. According to the 'six supergroups' model of eukaryotic phylogeny (Figure 1b) and other recent phylogenies, the separation of plants and animals/fungi could have been as early as the separation of any major groups of extant eukaryotes. Hence, gene duplications prior to the split of plants and animals/fungi can be placed at an early stage of eukaryotic evolution.

In this study, we included three representatives of Archaeplastida (the flowering plant *Arabidopsis thaliana*, the moss *Physcomitrella patens *and the green alga *Chlamydomonas reinhardtii*), three animals (*Homo sapiens*, the pufferfish *Takifugu rubripes *and the sea urchin *Strongylocentrotus purpuratus*) and two fungi (the budding yeast *Saccharomyces cerevisiae *and the fission yeast *Schizosaccharomyces pombe*), which all have nearly complete genome sequences (Table S1 in Additional file 1). According to a widely accepted model for the eukaryotic origin, the ancestral eukaryotic cell was derived from an Archaea-like organism, with additional genes originated from the endosymbiosis of a proteobacterium-like cell, which evolved into the mitochondrion [37]. Therefore, we included genes from three bacteria (*Escherichia coli*, *Rickettsia prowazekii *and *Bacillus subtilis*) and three archaea (*Methanosarcina acetivorans*, *Sulfolobus solfataricus *and *Pyrobaculum aerophilum*) as outgroups (Table S1 in Additional file 1).

The predicted protein sequences from all these 14 species were clustered using the Markov Clustering Algorithm (MCL; see Methods), which is among the most popular clustering methods and has been shown to be reliable [38]. By using a relatively low clustering stringency, 222,436 annotated protein sequences from the 14 representative species were divided into 51,396 gene clusters in total. Among these, 1,394 clusters contained both prokaryotic and eukaryotic genes and 41,444 clusters were eukaryote-specific. In addition, 794 out of the 1,394 clusters and 2,276 out of the 41,444 clusters contained genes from both Archaeplastida and Opisthokonta. The numbers of clusters of other phyletic patterns are summarized in Table S2 in Additional file 1.

### Analysis I - MCL clusters with both prokaryotic and eukaryotic genes

On the basis of the 794 clusters with genes from Archaeplastida, Opisthokonta, and prokaryotes, we retained only the clusters that had at least three eukaryotic genes, with at least one from Archaeplastida and at least one from Opisthokonta, as this is the minimum requirement for the deduction of a possible early eukaryotic duplication prior to the divergence of these two lineages. Also, to ensure the quality of these clusters, we tested the clusters by searching for one or more common domains in all members and subsequently removed sequences, if any, that lacked the most common domain(s) from each cluster. As a result, we obtained 772 gene clusters that meet these criteria and used them for phylogenetic analyses (Additional file 2). The phylogeny for each cluster was estimated by the neighbor-joining (NJ) method with bootstrap (BS) test and the maximum-likelihood (ML) method with BS and approximate likelihood ratio test (aLRT) (see Methods). The resulting tree topologies were then examined. Most gene families known to have experienced duplication in early eukaryotes were successfully recovered by our analysis (Table S3 in Additional file 1). Since our clusters were established based on sequence similarity instead of strict orthology, the eukaryotic genes in one cluster might be derived from more than one prokaryotic ancestor. To best distinguish the duplication in early eukaryotes from paralogy before the prokaryote-eukaryote separation, we identified orthogroups in each tree; each orthogroup consisted of eukaryotic genes that, most likely, originated from the same gene in the first eukaryotic common ancestor. According to the tree topology (Figure 1c), we defined an orthogroup as a eukaryotic clade that meets both of the following criteria: it has members from both plants and animals/fungi; and it has a prokaryotic outgroup (designated as type I orthogroups; for example, clades 1 and 2 in Figure 1c) or being a sister to another orthogroup that has a prokaryotic outgroup (designated as type II orthogroups; for example, clades 3 and 4 in Figure 1d). According to these criteria, we identified about 700 orthogroups. In each orthogroup, an ancient duplication event was inferred to be prior to the divergence of plants and animals/fungi if the tree topology of the orthogroup had two or more eukaryotic clades of which at least one clade consisted of members from both plants and animals/fungi. According to this definition, more than 35% (BS support ≥ 50%) or 20% (BS support ≥ 70%) of the 700 orthogroups showed one or more ancient duplication events (Table 1). Furthermore, the aLRT test of ML phylogenies produced even higher percentages of orthogroups with an early eukaryotic gene duplication at support levels of both 50% and 70% (Table 1).

**Table 1:** Number of orthogroups and early eukaryotic duplications identified in analysis I

	NJ-BS^a^		ML-BS		ML-aLRT^b^	
			
	≥ 50%	≥ 70%	≥ 50%	≥ 70%	≥ 50%	≥ 70%
Type I orthogroup with duplication	205 (**136**)	119 (**88**)	199 (**135**)	104 (**82**)	282 (**188**)	234 (**166**)
Type I orthogroup total	522	435	511	445	599	560
Type II orthogroup with duplication	100 (**63**)	61 (**43**)	72 (**46**)	37 (**29**)	81 (**60**)	85 (**66**)
Type II orthogroup total	235^c^	260	229^c^	234	176^c^	196
Total orthogroup with duplication	305 (**199**)	180 (**131**)	271 (**181**)	141 (**111**)	363 (**248**)	319 (**232**)
Orthogroup total	757	695	740	679	775	756
Percentage	40.3% (**26.3%**)	25.9% (**18.8%**)	36.6% (**24.5%**)	20.8% (**16.3%**)	46.8% (**32.0%**)	42.2% (**30.7%**)

Type I orthogroup refers to orthogroups with a prokaryotic outgroup; type II orthogroup refers to orthogroups without a prokaryotic outgroup. Entries in bold and in parentheses indicate that the duplications were inferred based on stringent criteria that required that at least one species was present in both paralogous clades. ^a^BS, bootstrap test. ^b^aLRT, approximate likelihood-ratio test. ^c^These numbers of type II orthogroups at a support level of ≥ 70% are greater than that at a support level of ≥ 50% since some type II orthogroups with ≥ 70% support were from type I orthogroups with ≥ 50% support whose prokaryotic outgroup had support less than 70%.

We reasoned that some of the gene duplications identified might be caused by long-branch attraction (LBA) artifacts in phylogenetic reconstruction. For example, in an orthogroup with the phyletic pattern of ((plants, animals, fission yeast) (budding yeast)), it was possible that the fission yeast gene evolved rapidly and was placed at the basal position due to LBA. In this case, a duplication event would be inferred based on the incorrect topology. Therefore, to minimize the impact of LBA, we used a more stringent criterion for the identification of gene duplication before the divergence of plants and animals/fungi: at least one gene from at least one species must be present in each of two paralogous clades. Based on this conservative criterion, we still found about 25% (BS ≥ 50%) or 15% (BS ≥ 70%) of the orthogroups to have experienced an early eukaryotic duplication (Table 1, entries in bold). Also, the ML-aLRT test showed that more than 30% of orthogroups (at support levels of both 50% and 70%) have experienced an early eukaryotic duplication (Table 1, entries in bold). This stringent criterion was also used in analyses II and III (see below). Moreover, we arbitrarily selected a subset of the orthogroups with topologies that were vulnerable to LBA, and added sequences from additional species to further test the impact of LBA. The results showed that phylogenies of most of the orthogroups tested (15 out of 21) still supported early eukaryotic duplication (Table S4 in Additional file 1). Especially, all six orthogroups that initially showed duplication at a support level of 70% still supported early eukaryotic duplication after adding more sequences. These results suggest that our phylogenetic topologies are quite reliable.

**Figure 2:**
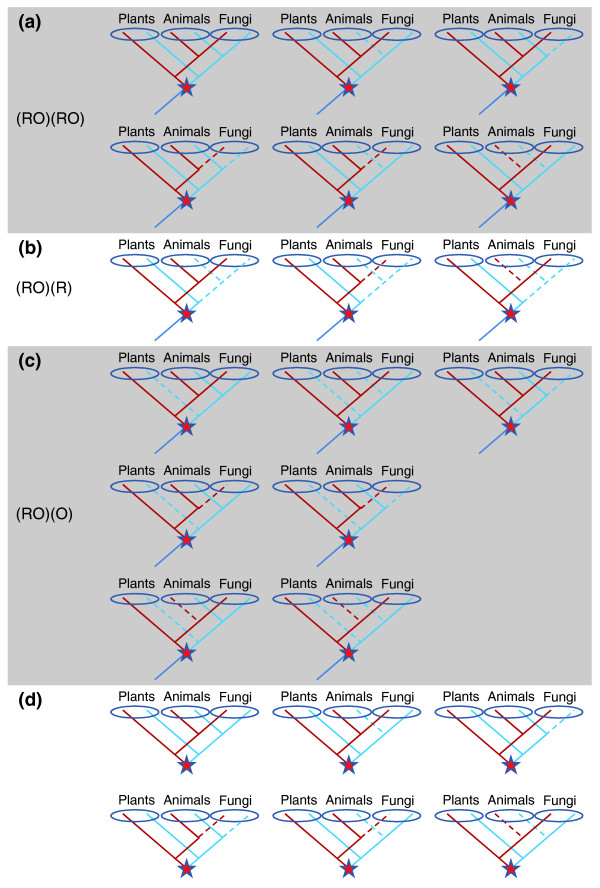
**Hypothetical examples of phylogenetic trees showing the patterns of retention of duplicates**. **(a) **Six phyletic patterns showing the (RO)(RO) pattern (both of the duplicates were retained in plants and animals/fungi). **(b) **Three phyletic patterns showing the (RO)(R) pattern (one of the duplicates was lost in animals/fungi). (c) Seven phyletic patterns showing the (RO)(O) pattern (one of the duplicates was lost in plants). (d) Six phyletic patterns that supported an early eukaryotic duplication in eukaryote-specific gene clusters.

To learn about the fate of the ancient duplicates, we also examined whether specific duplicates were retained or lost, and found that different orthogroups varied in their patterns of retention of duplicates. One possible fate was that both of the duplicates were retained in plants and animals/fungi (Figure 2a), abbreviated here as (RO)(RO) (R, Archaeplastida; O, Opisthokonta). Among all the orthogroups that showed early eukaryotic duplication, about 35% displayed this pattern (Table 2). Alternatively, one of the duplicates could be lost in either plants or animals/fungi, abbreviated here as (RO)(R) and (RO)(O), respectively (Figure 2b, c). These two topologies were less frequent than (RO)(RO) (Table 2). Similar results were obtained with different phylogenetic methods and at different levels of support. A small number of remaining orthogroups had more complex patterns (Table 2, 'Other' column), possibly due to multiple rounds of duplication and gene loss. The detailed distribution of phyletic patterns is summarized in Table S5 in Additional file 1.

**Table 2:** Distribution of orthogroups with phyletic patterns supporting early eukaryotic duplication

Dataset	Method	Support	(RO)(RO)	(RO)(R)	(RO)(O)	Other^a^	Total
Analysis I	NJ-BS^b^	≥ 50%	73 (36.7%)	56 (28.1%)	59 (29.6%)	11 (5.5%)	199
		≥ 70%	52 (39.7%)	31 (23.7%)	34 (26.0%)	14 (10.7%)	131
							
	ML-BS	≥ 50%	71 (39.2%)	55 (30.4%)	46 (25.4%)	9 (5.0%)	181
		≥ 70%	46 (41.4%)	29 (26.1%)	21 (18.9%)	15 (13.5%)	111
							
	ML-aLRT^c^	≥ 50%	102 (41.1%)	75 (30.2%)	64 (25.8%)	7 (2.8%)	248
		≥ 70%	95 (40.9%)	63 (27.2%)	62 (26.7%)	12 (5.2%)	232
							
Analysis III	NJ-BS	≥ 50%	90 (30.9%)	72 (24.7%)	94 (32.3%)	35 (12.0%)	291
		≥ 70%	40 (26.3%)	41 (27.0%)	41 (27.0%)	30 (19.7%)	152
							
	ML-BS	≥ 50%	92 (33.9%)	80 (29.5%)	62 (22.9%)	37 (13.7%)	271
		≥ 70%	39 (30.2%)	33 (25.6%)	22 (17.1%)	35 (27.1%)	129
							
	ML-aLRT	≥ 50%	299 (48.3%)	156 (25.2%)	156 (25.2%)	8 (1.3%)	619
		≥ 70%	268 (46.4%)	136 (23.6%)	150 (26.0%)	23 (4.0%)	577

^a^All the orthogroups for which the pattern of retention of duplicates cannot be explicitly determined are assigned to the 'Other' category. ^b^BS, bootstrap test. ^c^aLRT, approximate likelihood-ratio test. R, Archaeplastida; O, Opisthokonta; (RO)(RO), both duplicates were retained in plants and animals/fungi; (RO)(O), one of the duplicates was lost in plants; (RO)(R), one of the duplicates was lost in animals/fungi.

**Figure 3:**
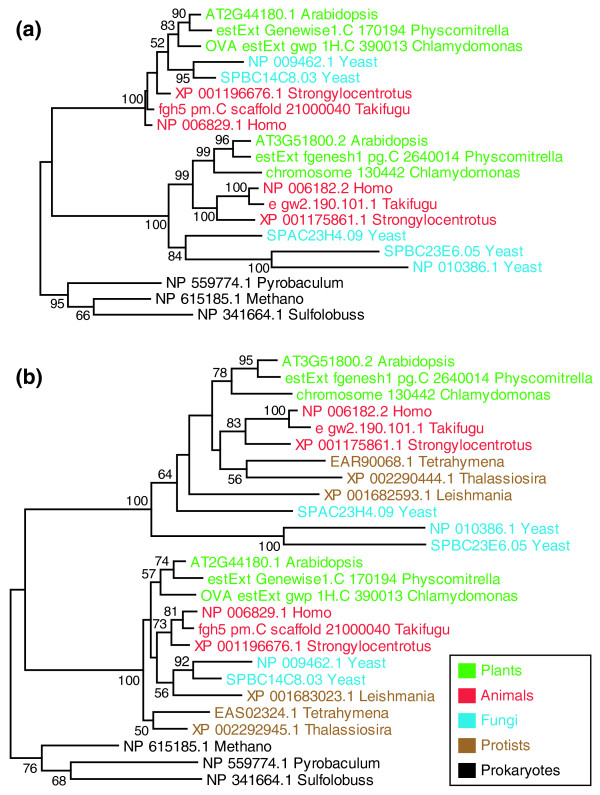
**Exemplar phylogenetic tree of an orthogroup (Cluster_212) with early eukaryotic duplication**. **(a) **Topology of the ML tree, showing this orthogroup had experienced duplication before the plants-animals/fungi split. **(b) **Topology of the ML tree with protist sequences, showing the duplication happened before the divergence of 'early branching' protists.

In the context of the 'six supergroups' model of eukaryotic evolution (Figure 1b), the gene duplications we identified were very ancient events as they happened before the separation of Archaeplastida and Opisthokonta. This split possibly represents the most ancient eukaryotic divergence among extant groups. However, the 'crown-stem' model (Figure 1a) suggests that the plants-animals/fungi split is relatively recent in comparison to several 'early branching' protists, such as members of Excavata and Chromalveolata. To further place the duplications we identified, we added sequences from representative 'early branching' protists (Excavata: *Giardia lamblia*, *Trichomonas vaginalis*, *Trypanosoma brucei *and *Leishmania major*; Chromalveolata: *Plasmodium falciparum *and *Phaeodactylum tricornutum*; Amoebazoa: *Dictyostelium discoideum *and *Entamoeba histolytica*) to orthogroups with duplication (identified by the ML method at a BS ≥ 70% support level). Additional protists (for example, Chromalveolata: *Tetrahymena thermophila*, *Paramecium tetraurelia *and *Toxoplasma gondii*) were searched if no homolog could be found in the previous group of representative species. We found that most (84 out of 111) of the orthogroups had protist sequences in at least one of the paralogous clades (see Figure 3, for example; see Additional file 2 for details). Among the remaining 27 orthogroups, 19 orthogroups had no resolution, 2 orthogroups had no detectable protist homologs and only 6 orthogroups supported a different phylogeny that placed the duplication after the divergence of early protists from animals/plants. These results strongly suggest that most of these duplications were indeed very ancient events, regardless of which eukaryotic phylogenetic model ('crown-stem' or 'six supergroups') was used.

### Analysis II - MCL clusters with eukaryotic genes only

Because analysis I required that each cluster contain some prokaryotic gene(s), the total number of gene clusters was limited. To more widely represent the eukaryotic genomes in our study, we examined gene clusters that contained only eukaryotic genes. Among the 41,444 eukaryote-specific gene clusters (Table S2 in Additional file 1), 2,276 clusters contain members from both plants and animals/fungi, suggesting that they are likely descendants of ancestral genes in the early eukaryotes. Therefore, the phylogenies of these clusters could also provide evidence for early eukaryotic duplication. Due to the lack of prokaryotic outgroups, it was difficult to determine the root for the phylogeny of a eukaryote-specific cluster. However, a duplication event could still be unambiguously inferred if a bipartition could be found in the tree in which both portions had sequences from plants and animals/fungi (see Figure 1d for an illustration). This means that the cluster should have at least two sequences from each of the plant and animal/fungal lineages. After filtering out sequences that lack common domains, 1,903 clusters met this criterion and were further investigated by phylogenetic analysis (Additional file 2). The results show that, even at a support level of 70%, more than 10% of the clusters exhibit evidence of duplication before the separation of plants and animals/fungi (Table 3).

**Table 3:** Number of orthogroups and early eukaryotic duplications identified in analysis II

Method	Support	Number of orthogroups with duplication	Percentage out of 1,903 clusters
NJ-BS^a^	≥ 50%	275	14.5%
	≥ 70%	216	11.4%
			
ML-BS	≥ 50%	248	13.0%
	≥ 70%	194	10.2%
			
ML-aLRT^b^	≥ 50%	304	16.0%
	≥ 70%	283	14.9%

^a^BS, bootstrap test. ^b^aLRT, approximate likelihood-ratio test.

### Analysis III - reanalysis of the KOG-to-COG clusters

To further strengthen our investigation of ancient eukaryotic gene duplication, we wanted to test an independent dataset of gene clusters to evaluate the reliability of the results. We used an existing dataset of gene clusters with both eukaryotic and prokaryotic members that was established with a different methodology from that of our analysis I [36]; this is our analysis III. In their study, Makarova *et al*. [36] used established databases [39] of prokaryotic clusters of orthologous groups (COGs) and their eukaryotic counterparts (KOGs) to construct KOG-to-COG clusters. A COG was defined by best hits from BLAST analyses with members from at least three relatively distant prokaryotes among a total of 63 species included in the study [39]. Similarly, a KOG contains best hits from at least three eukaryotic species from a group of seven in the earlier study [39]; the total number of eukaryotes was increased to 11 subsequently [36]. The authors used RPS-BLAST search to find the best COG hit for each KOG and all the KOGs that have the same COG best-hit were assigned to one cluster [36]. In total, they identified 1,092 KOG-to-COG clusters (each with one COG), which covered 2,445 KOGs [36] (Additional file 2).

Since the KOG database does not include some of the representative species used in analysis I, we first assigned the predicted protein sequences from *Physcomitrella*, *Chlamydomonas*, *Takifugu *and *Strongylocentrotus *to KOGs. Then, we extracted the sequences of the 14 representative species from each KOG-to-COG cluster, and retained only the clusters that had at least one prokaryotic gene and three eukaryotic genes, with at least one from plants and one from animals/fungi. As a result, 89 out of the 1,092 KOG-to-COG clusters were excluded from further analysis due to their failure to meet the criteria. The phylogenies for the remaining 1,003 clusters were estimated by using both NJ and ML methods. The same criteria as used in analysis I were followed to identify orthogroups and infer early eukaryotic gene duplication. As summarized in Table 4, while the total number of orthogroups (about 900 at a BS ≥ 70% support level) was higher, the percentages of orthogroups with early eukaryotic duplication we observed were similar to those from analysis I. Much higher percentages (more than 40%) of orthogroups with an early eukaryotic duplication were suggested by the ML-aLRT test at support levels of both 50% and 70% (Table 4). The distribution of orthogroups with different phyletic patterns was also similar to analysis I (Table 2; Table S6 in Additional file 1).

**Table 4:** Number of orthogroups and early eukaryotic duplications identified in analysis III

	NJ-BS^a^		ML-BS		ML-aLRT^b^	
			
	≥ 50%	≥ 70%	≥ 50%	≥ 70%	≥ 50%	≥ 70%
Type I orthogroup with duplication	172	93	169	80	334	276
Type I orthogroup total	508	389	526	380	774	680
Type II orthogroup with duplication	119	59	102	49	285	301
Type II orthogroup total	724	597	605	504	581	659
Total orthogroup with duplication	291	152	271	129	619	577
Orthogroup total	1,232	986	1,131	884	1,355	1,339
Percentage	23.6%	15.4%	24.0%	14.6%	45.7%	43.1%

Type I orthogroup refers to orthogroups with a prokaryotic outgroup; type II orthogroup refers to orthogroups without a prokaryotic outgroup. ^a^BS, bootstrap test. ^b^aLRT, approximate likelihood-ratio test.

### Comparison of gene copy number between human and *Arabidopsis*

**Figure 4:**
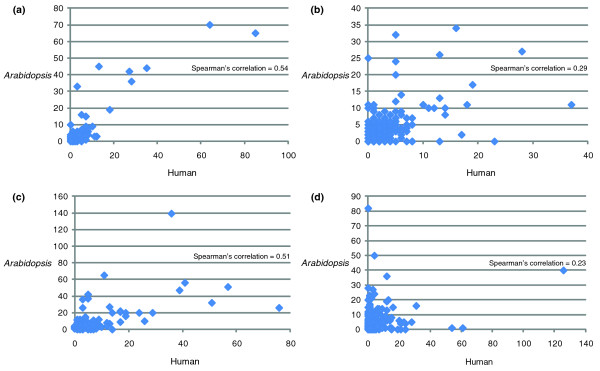
**Comparison of gene copy number between human and *Arabidopsis***. The gene copy number of each family (ML approach, BS ≥ 70) in human versus that in *Arabidopsis *was plotted. **(a) **Families with prokaryotic outgroups and early eukaryotic duplication. **(b) **Families with prokaryotic outgroups but no early eukaryotic duplication. **(c) **Families without prokaryotic outgroups but show early eukaryotic duplication. **(d) **Families without prokaryotic outgroups nor early eukaryotic duplication. The differences between Spearman correlation coefficients for both (a) versus (b) and (c) versus (d) are statistically significant (*P*-value < 0.01). The statistical significances were obtained through permutation test.

Many gene families have experienced duplication during the evolution of plants or animals, and gene copy can either remain similar or differ dramatically between organisms [30,31,33,40,41], possibly related to functional evolution. To further investigate the properties of families in our studies that showed detectable gene duplication before the animal-plant split, versus the families that did not have such duplications, we plotted the gene copy number of each family in human versus that in *Arabidopsis *and calculated the Spearman's correlation coefficients (Figure 4). We found that among the families that had a prokaryotic outgroup, those that exhibited the early eukaryotic duplication showed a positive correlation of gene copy number between human and *Arabidopsis *(Figure 4a), whereas the families that did not have detectable early duplication had a much less positive correlation between human and *Arabidopsis *(Figure 4b). The difference between the two correlation coefficients was significant (*P*-value < 0.01), according to the permutation test. Similarly, for the families that did not have a prokaryotic outgroup, the families with an early duplication showed a significantly stronger positive correlation than the families without the duplication (Figure 4c, d).

## Discussion

### Detection of very ancient eukaryotic gene duplications

In this study, we investigated the extent of eukaryotic gene duplication before the divergence of plants and animals/fungi by constructing gene clusters with members from representative prokaryotic and eukaryotic species and performing comprehensive phylogenetic analyses.

As we sampled only a small number of species from each lineage, additional cluster analyses were performed by adding genes from zebrafish (teleost fish), medaka (teleost fish), *Drosophila melanogaster *(insect) or the giant clam *Lottia gigantean *(mollusc), respectively (see Additional file 3 for complete clustering results). We found that adding genes from each of the additional species resulted in very slight changes in gene cluster numbers (Table S7 in Additional file 1). Therefore, we believe that our overall results would not be dramatically affected by inclusion of the additional animal species.

Our analysis I was based on the gene clusters delineated by the MCL method, and revealed that about 25% (BS ≥ 50%) or 15% (BS ≥ 70%) of orthogroups had experienced ancient gene duplication. Higher numbers and percentages of orthogroups that showed ancient gene duplication were reported by the ML-aLRT test (also in analyses II and III), possibly because the bootstrap test is consistently conservative [42]. It is known that, in comparative genomics studies like the ones we performed here, the accuracy of gene family clustering has a great impact on the reliability of subsequent analyses such as phylogenetic reconstruction. Therefore, it is of interest to check whether alternative strategies of gene family clustering would lead to similar results as the MCL approach used in analysis I. COG and its eukaryotic equivalent, KOG, are among the most widely used databases of orthologous gene clusters. In our analysis III, we took the KOG-to-COG clusters identified by Makarova *et al*. [36] and analyzed them using the same procedures as used in analysis I. In comparison to analysis I, in analysis III we obtained a very similar percentage of orthogroups showing early eukaryotic duplication, although the total number of orthogroups identified was higher. Interestingly, however, we found that less than half of the orthogroups with duplication overlap between the two analyses. The differences were mainly due to two reasons: first, the prokaryotic members in a particular MCL cluster were not in any COG or the corresponding COG were not in any KOG-to-COG cluster; second, a KOG-to-COG cluster may include sequences of very limited similarity, resulting in a phylogeny different from that of the corresponding MCL cluster. Nonetheless, the fact that different gene family clustering methods (MCL and COG/KOG) and phylogenetic approaches (NJ and ML) all revealed similar percentages of orthogroups that had experienced early eukaryotic duplication still supports the reliability of our results.

One possible bias in our analysis I is that only the eukaryotic genes with detectable prokaryotic homologs were studied. This means that we focused on relatively conserved genes. In consideration of the antiquity of the gene duplication events we are interested in, some eukaryotic genes might lack detectable homologs in the prokaryotes in our study due to gene loss or sequence divergence and thus were not included in our analysis I. For this reason, we also carried out analysis II to analyze the eukaryote-specific MCL gene clusters and found that more than 10% of the 1,903 gene clusters showed early eukaryotic duplication. It is possible that this figure is still an underestimation since some of the ancient duplicates might fail to be clustered together due to a high degree of divergence and would appear as separate gene clusters without early eukaryotic duplication.

Our phylogenetic analyses identified approximately 300 (BS support ≥ 70%) or approximately 500 (aLRT support ≥ 70%) gene duplications in the time window from the origin of eukaryotes to the plants-animals/fungi split. However, the estimation of the length of this time window varies depending on which eukaryotic phylogeny is adopted. According to the 'crown-stem' model of eukaryotic phylogeny (Figure 1a), plants and animals/fungi are members of a crown group and several groups of protists form deep branches in the tree [18,19]. It was estimated that plants and animals/fungi separated approximately 1,600 million years ago (MYA), and *Giardia*, which was considered the deepest branch in the eukaryotic tree of life, diverged approximately 2,300 MYA [43]. Given the estimated origin of eukaryotes at approximately 2,700 MYA [44], the duplication events identified in our study could have taken place during the long time period before the separation of plants and animals/fungi (approximately 1,100 million years). A contrasting picture is depicted by the more recent 'six supergroups' classification of eukaryotes (Figure 1b) [21-23].

In this model and other related models, both the 'unikont-bikont' topology [26,27] and the recent 'photosynthetic-nonphotosynthetic' bipartition [29] suggest that the Archaeplastida-Opisthokonta separation might represent the first major split, or at least one of the early splits, in eukaryotic evolution (Figure 1b). In this perspective, the duplication events we identified could be placed during a very early stage of eukaryotic evolution, prior to the divergence of most of the major extant protist groups.

Regardless of whether the 'crown-stem' model, or 'six supergroups' and other similar models are correct, we investigated gene duplications among a wider representation of eukaryotes using phylogenetic analyses with additional sequences from exemplars of divergent major protist groups, Excavata, Amoebozoa, and Chromalveolata (Figure 1b). For most of the gene families with 70% BS support, the duplication likely occurred prior to the separation of these highly divergent protists from plants and/or animals/fungi. Even according to the 'crown-stem' model of early eukaryotic history, these divergent protists separated from plants/animals/fungi at an earlier time. Therefore, irrespective of the models of early eukaryotic phylogeny, these duplications would be placed before any known major eukaryotic divergence. Therefore, our results support many gene duplication events during very early eukaryotic evolution.

### Functional implication for early eukaryotic evolution

The gene duplications we detected likely generated raw materials for functional evolution, as proposed before [4]. Indeed, the duplicates from the 300 or more gene duplications we identified would most likely be eliminated if they did not provide selective advantage. Therefore, these early eukaryotic gene duplications could have been of great importance for the success and radiation of early eukaryotes, and thus have been retained in the last common ancestor of extant major eukaryotic groups. If the duplicated gene families are involved in processes that are fundamental to early eukaryotes, which are likely to be also shared by extant eukaryotes, they might show similar evolutionary patterns in different eukaryotic kingdoms. Specifically, copy numbers for genes with highly conserved functions seem to be more stable than the number of genes with more divergent functions (compare *RAD51*, *MSH*, and *SMC *with *JmjC *and MADS-box genes) [30,31,33-35].

In fact, we observed a more positive correlation of gene family size between animals and plants in the families with early eukaryotic duplication than in the families without such duplication (Figure 4). In other words, the families with the early eukaryotic duplication tend to have more similar evolutionary patterns in both plants and animals/fungi than those families without the early duplication, suggesting that these genes might have relatively conserved functions among the three major kingdoms. This idea of functional conservation is also supported by the finding that the (RO)(RO) pattern, in which both duplicates are retained in both the plants and animal/fungi lineages, is the most frequent pattern among all possible patterns.

Also, it is of interest to know whether genes with specific biochemical or molecular functions or involved in specific processes are enriched among the families with duplication. Interestingly, our Gene Ontology (GO) analysis did not reveal any GO terms significantly enriched among the orthogroups with duplication (data not shown). This might suggest that the detected gene duplications, which we propose could have benefited the early eukaryotic ancestor and the ancestors of both the plant and animal/fungi lineages, affected many types of functions and processes, not just a few specialized classes of functions.

### A hypothesis for early eukaryotic large-scale duplication

Gene duplication can be generated by several mechanisms, including tandem duplication, transposition and large-scale duplication (for example, segmental/whole genome duplication (WGD)). In principle, the 300 or more gene duplications we identified could be independent events resulting from tandem duplication and transposition. However, in the absence of supporting evidence, such a complex pattern of multiple independent events is not parsimonious. Alternatively, the duplications could be explained by one or a few large-scale duplications. Large-scale duplication, like WGD, is of special interest because it allows the generation of multiple new functional modules with many genes that are unrelated at the sequence level [45], which would not be likely by other duplication mechanisms. Also, segmental duplications (SDs) are increasingly recognized as frequent phenomena, especially in primate genomes - for example, approximately 5% of the human genome consists of duplicated segments [46]. Therefore, SDs with sufficiently large numbers of genes could also account for the gene duplications we detected. After WGD/SDs, the different fates of duplicated genes in different populations could generate the genetic diversity that then allows both reproductive isolation/speciation and environmental adaptation [47,48].

The large number of ancient eukaryotic duplication events that we have detected here could have been the result of one or more early eukaryotic large-scale duplications. For relatively recent large-scale duplication events, it is possible to identify syntenic genomic regions [49]. For example, such syntenic regions were found for the most recent WGD in *Arabidopsis*, poplar and yeast, which likely occurred approximately 100 MYA or more recently [10-12,50]. However, for older ones such as the WGDs in vertebrate (1R/2R; approximately 525 to 875 MYA [51]), synteny is no longer detectable due to numerous genome rearrangements and gene loss [52]. If a large-scale duplication was the cause of the ancient gene duplication events identified in this study, this event would have occurred at least 1,600 MYA (possibly even earlier), making it exceedingly unlikely that any synteny can still be detected. Another approach to the detection of large-scale duplication is to analyze the rate of synonymous base substitutions (dS) between paralogous genes, as reported for many plant species [53,54]. Unfortunately, this method is also not feasible for events older than approximately 150 million years because of the saturation of dS values.

An alternative way to obtain evidence for large-scale duplication is to examine the phylogeny of a large number of gene families, as we have done here. Our results indicate that a significant fraction of the orthogroups in our dataset had experienced duplication before the divergence of the three major eukaryotic kingdoms. By combining the results of analyses I and II, we estimated that the percentage of orthogroups showing duplication before the separation of plants and animals/fungi is over 15% (BS ≥ 50% support level) and 10% (BS ≥ 70% support level), or about 30% (aLRT support ≥ 50%) and 20% (aLRT support ≥ 70%). Similar large-scale phylogenetic analyses showed that, among the duplicate pairs resulting from more recent WGD in vertebrates (1R/2R; approximately 525 to 875 MYA) and yeast (approximately 100 MYA), 26.6% and 20.1% of the pairs survived, respectively [51,55]. The early eukaryotic duplications we studied were much more ancient than the previously reported large-scale duplications in animals, plants and yeast. Thus, during the at least 1,600 million years of evolution, the duplicate pairs that arose in early eukaryotes might have had a higher chance to be lost or to be too divergent to be recognized. Therefore, it is reasonable to expect that a lower percentage of the duplicate pairs would survive, and our phylogenetic results could support the hypothesis that the duplication events identified here are the remnants of a large-scale duplication (for example, WGD or SDs) in early eukaryotes. In other words, considering the antiquity of the early eukaryotic duplications, the 300 or more duplications we detected probably represent only a small fraction of the real number of duplications in early eukaryotes, which could be in the thousands. Our results could be most parsimoniously interpreted by one or more large-scale duplications, which were likely to be WGD/SDs, rather than thousands of independent duplications.

## Conclusions

In this study, we conducted extensive phylogenetic analyses to investigate the extent of gene duplication in early eukaryotic evolution. We have found at least 300 orthogroups that had likely experienced an ancient eukaryotic duplication event prior to the divergence of the major eukaryotic supergroups. Our results provide a better understanding of early eukaryotic evolution in several ways. The identification of numerous ancient eukaryotic gene duplication events suggests that gene duplication played an important role in the evolution of early eukaryotes. The large number of duplicated genes might have allowed large-scale evolution of new gene functions, increasing the chance of greater species diversity in changing environments. In particular, the shared duplications in plants and animals/fungi might have contributed to the three independent origins of multicellularity in these lineages. Furthermore, these ancient duplications could be most simply explained by a hypothesized early eukaryotic WGD/SDs. We further postulate that this/these WGD/SDs might have contributed to the early eukaryotic radiation. Therefore, like the early vertebrate and angiosperm diversifications, the hypothesized WGD/SDs could provide an explanation at the level of genome evolution for the high rate of speciation near the origin of the three major eukaryotic lineages.

## Materials and methods

### Reconstruction of gene clusters

For analyses I and II, the predicted protein sequences of the 14 representative species were retrieved from public databases (see Table S1 in Additional file 1 for the complete list of data sources). These protein sequences were compared using an all-to-all BLASTP search with a cut-off of 1e^-10 ^[56]. Based on the BLASTP results, MCL clustering was performed with low stringency (inflation value of 1.5) to produce gene clusters [38]. To check the clusters for common domains, the domain architectures of all cluster members were annotated using InterProScan v4.5 (InterPro release 22.0, including both integrated and un-integrated) [57].

For analysis III, we started from the 1,092 KOG-to-COG clusters identified in the study of Makarova *et al*. [36]. Since the original KOG database does not cover the genomes of *Physcomitrella*, *Chlamydomonas*, *Takifugu *and *Strongylocentrotus*, the predicted protein sequences from these four species were assigned to KOGs using BLASTP search. Then the sequences from the 14 representative prokaryotic and eukaryotic species were extracted from each KOG-to-COG cluster to form the dataset for the following phylogenetic analysis.

### Phylogenetic analysis

For all the MCL gene clusters and KOG-to-COG clusters, highly similar sequences (more than 80% identity) from the same species were removed by using BLASTCLUST [56]. Multiple sequence alignments were generated by using MUSCLE 3.6 [58]. The multiple sequence alignments were trimmed by removing poorly aligned regions using trimAl 1.2 with the automated1 option [59]. NJ trees were constructed using PHYLIP 3.68 (JTT model) with 1,000 bootstrap replicates [60,61]. ML trees were constructed using RAxML 7.2.0 (LG model plus gamma correction) with 100 bootstrap replicates [62,63]. The best-scoring ML trees were also evaluated with the aLRT method by using Phyml 3.0 [64,65]. For large clusters with more than 100 sequences, representative sequences were selected based on a preliminary NJ tree. Phylogenetic trees were screened by custom scripts to identify orthogroups and duplication events. All scripts in this study, gene clusters and phylogenetic trees are available upon request.

### Gene Ontology analysis

Orthogroups with early eukaryotic duplication were compared with orthogroups that did not have such duplications for overrepresented GO terms [66]. Domains encoded by the majority of orthogroup members were considered representatives for the orthogroup. Then GO annotations of representative InterPro domains were assigned to each orthogroup using InterPro2GO mapping [67]. Subsequently, all GO annotations were mapped to GO slims, a cut-down version of GO, using the map2slim perl script and generic GO slim version 1.2 [67]. The overrepresentation of GO slims was examined using Ontologizer 2.0 [68] with term-for-term analysis and Bonferroni correction for multiple testing.

## Abbreviations

aLRT: approximate likelihood ratio test; BS: bootstrap; COG: prokaryotic clusters of orthologous groups; GO: Gene Ontology; KOG: eukaryotic clusters of orthologous groups; LBA: long-branch attraction; MCL: Markov Clustering Algorithm; ML: maximum-likelihood; MYA: million years ago; NJ: neighbor-joining; SD: segmental duplication; WGD: whole genome duplication.

## Authors' contributions

XZ performed the analyses and drafted the manuscript. ZL contributed to the analysis of the KOG-to-COG clusters and the analysis of protist sequences and commented on the manuscript. HM conceived of and supervised the study and critically revised the manuscript. All authors read and approved the final manuscript.

## Supplementary Material

Additional file 1 **Supplemental Tables S1 to S7**. Table S1: a summary of representative species included in this study. Table S2: a summary of MCL gene clustering results. Table S3: a summary of gene families known to have experienced early eukaryotic gene duplication. Table S4: test of the impact of long-branch attraction on orthogroups with vulnerable topologies. Table S5: distribution of orthogroups with phyletic patterns supporting early eukaryotic duplication - analysis I. Table S6: distribution of orthogroups with phyletic patterns supporting early eukaryotic duplication - analysis III. Table S7: results of MCL clustering analyses with genes from additional animal species. Click here for file

Additional file 2 **Information about all the gene clusters analyzed in analyses I, II and III, including gene cluster ID, accession number for each cluster member and information about whether the cluster exhibit early eukaryotic duplication with different phylogenetic methods and bootstrap support levels**. In addition, for gene clusters analyzed with additional sequences from divergent protists, information about the protist species included in each cluster and the phyletic pattern is provided. Click here for file

Additional file 3 **Information about MCL clustering analyses with genes from additional animal species, including gene cluster ID and accession number of each cluster member**. The gene clusters are also cross-referred to the clusters analyzed in analysis I/II and labeled with one of the following terms; 'same' - the new cluster contains the same members as the cluster analyzed in snalysis I/II, except for the genes from additional species; 'parent_set' - the new cluster contains all the genes in the cluster analyzed in analysis I/II, but not 'same'; 'subset' - all genes in the new cluster (except for genes from additional species) are included in the cluster analyzed in analysis I/II, but not 'same'; 'overlapping' - more than 50% genes in the new cluster (except for genes from additional species) are included in the cluster analyzed in analysis I/II, but not among the previous three types. Click here for file
