# Supplementary material for: Phylogenetic detection of numerous gene duplications shared by animals, fungi and plants
Source: Genome Biol. 2010 Apr 6;11(4):R38. doi: 10.1186/gb-2010-11-4-r38 (PMC2884541; doi:10.1186/gb-2010-11-4-r38)
Supplement: Additional file 1 — Supplemental Tables S1 to S7. Table S1: a summary of representative species included in this study. Table S2: a summary of MCL gene clustering results. Table S3: a summary of gene families known to have experienced early eukaryotic gene duplication. Table S4: test of the impact of long-branch attraction on orthogroups with vulnerable topologies. Table S5: distribution of orthogroups with phyletic patterns supporting early eukaryotic duplication - analysis I. Table S6: distribution of orthogroups with phyletic patterns supporting early eukaryotic duplication - analysis III. Table S7: results of MCL clustering analyses with genes from additional animal species. [file gb-2010-11-4-r38-S1.DOC]

**Table S1:** Summary of representative species included in this study

|  | Species | Data source | Predicted proteins | Analyzed proteins* |
| --- | --- | --- | --- | --- |
| Plants | *Arabidopsis thaliana*  (Flowering plant) | TAIR Version 8  (www.arabidopsis.org) | 32825 | 13299 |
|  | *P*hyscomitrella patens  (Moss) | JGI Version 1.1  (ftp.jgi-psf.org/pub/JGI_data/) | 35938 | 9517 |
|  | Chlamydomonas reinhardtii  (Green algae) | JGI Version 4.0  (ftp.jgi-psf.org/pub/JGI_data/) | 16709 | 4697 |
| Animals | *Homo sapiens*  (Human) | Build 36.3  (ftp.ncbi.nih.gov/genomes/) | 37742 | 11830 |
|  | *Takifugu rubripes*  (Pufferfish) | JGI Version 4.0  (ftp.jgi-psf.org/pub/JGI_data/) | 26721 | 9205 |
|  | *Strongylocentrotus purpuratus*  (Sea urchin) | Build 2.1  (ftp.ncbi.nih.gov/genomes/) | 42420 | 18495 |
| Fungi | *Saccharomyces cerevisiae*  (Budding yeast) | Build 2.1  (ftp.ncbi.nih.gov/genomes/) | 5861 | 2872 |
|  | *Schizosaccharomyces pombe*  (Fission yeast) | 06Aug2008  (ftp.sanger.ac.uk/pub/yeast/) | 5026 | 2844 |
| Eubacteria | *Escherichia coli str. K-12 substr. MG1655*  (Gram-negtive bacteria) | (ftp.ncbi.nih.gov/genomes/Bacteria/) | 4132 | 953 |
|  | *Rickettsia prowazekii str. Madrid E*  (Gram-negtive bacteria) | (ftp.ncbi.nih.gov/genomes/Bacteria/) | 835 | 277 |
|  | *Bacillus subtilis subsp. subtilis str. 168*  (Bacilli) | (ftp.ncbi.nih.gov/genomes/Bacteria/) | 4105 | 894 |
| Archaea | *Methanosarcina acetivorans C2A* | (ftp.ncbi.nih.gov/genomes/Bacteria/) | 4540 | 720 |
|  | *Sulfolobus solfataricus P2* | (ftp.ncbi.nih.gov/genomes/Bacteria/) | 2977 | 707 |
|  | *Pyrobaculum aerophilum str. IM2* | (ftp.ncbi.nih.gov/genomes/Bacteria/) | 2605 | 516 |

*: Analyzed proteins are the proteins included in MCL clusters analyzed in Analysis I and II.

**Table S2:** Summary of MCL gene clustering results

| Eukaryotes | | Prokaryotes | Number of clusters |
| --- | --- | --- | --- |
| Archaeplastida | Opishtokonts |
| + | + | + | 794 |
| + | - | + | 443 |
| - | + | + | 157 |
| + | + | - | 2276 |
| + | - | - | 21874 |
| - | + | - | 17294 |
| - | - | + | 8558 |
| Total | | | 51396 |

**Table S3:** Summary of gene families known to have experienced early eukaryotic gene duplication

| Gene family | Reference | Gene cluster | Pattern |
| --- | --- | --- | --- |
| SMC | (Surcel et al. 2008) | Analysis I: OG_101 | (RO)(RO) |
| recA | (Lin et al. 2006) | Analysis I: OG_102 | (RO)(RO) |
| MutS | (Lin et al. 2007) | Analysis I: OG_67 | (RO)(RO) by ML-aLRT / (RO)(R) by NJ |
| MutL | (Lin et al. 2007) | Analysis I: OG_100 | (RO)(RO) |
| Spo11 | (Malik et al. 2007) | Analysis I: OG_426 | (RO)(R) by ML-BS and NJ / (AO)(AO) by ML-aLRT |
| Kinesin | (Miki et al. 2005) | Analysis II: OG_10 | (RO)(RO) |
| MADS | (Alvarez-Buylla et al. 2000) | The paralogous clades divided into separate gene clusters (Type-I - Analysis II: OG_52) | No duplication |
| KDM1 | (Zhou and Ma 2008) | Analysis II: OG_143 | (RO)(RO) |
| JmjC | (Zhou and Ma 2008) | Analysis II: OG_426 (PKDM11 and JMJD6) | (RO)(RO) |
| RDRP | (Zong et al. 2009) | The paralogous clades do not cover the representative species used in this study |  |
| RNA Pol II | (Archambault and Friesen 1993) | Analysis I: OG_64 | (RO)(R) by ML-aLRT |
| DNA Pol | (Filee et al. 2002) | Analysis I: OG_142 | (RO)(RO) |
| MCM | (Kearsey and Labib 1998) | Analysis I: OG_53 | (RO)(RO) |
| TCP1 | (Gupta 1995) | Analysis I: OG_26 | (RO)(O) by ML-BS / (RO)(RO) by ML-aLRT |
| Proteasome subunits | (Hughes 1997) | Analysis I: OG_29 | (RO)(RO) by ML-aLRT |

R – Archaeplastida; O – Opishtokonta.

**References:**

Alvarez-Buylla, E.R., Pelaz, S., Liljegren, S.J., Gold, S.E., Burgeff, C., Ditta, G.S., Ribas de Pouplana, L., Martinez-Castilla, L., and Yanofsky, M.F. 2000. An ancestral MADS-box gene duplication occurred before the divergence of plants and animals. *Proc Natl Acad Sci* **97**: 5328-5333.

Archambault, J. and Friesen, J.D. 1993. Genetics of eukaryotic RNA polymerases I, II, and III. *Microbiol Rev* **57**: 703-724.

Filee, J., Forterre, P., Sen-Lin, T., and Laurent, J. 2002. Evolution of DNA polymerase families: evidences for multiple gene exchange between cellular and viral proteins. *J Mol Evol* **54**: 763-773.

Gupta, R.S. 1995. Evolution of the chaperonin families (Hsp60, Hsp10 and Tcp-1) of proteins and the origin of eukaryotic cells. *Mol Microbiol* **15**: 1-11.

Hughes, A.L. 1997. Evolution of the proteasome components. *Immunogenetics* **46**: 82-92.

Kearsey, S.E. and Labib, K. 1998. MCM proteins: evolution, properties, and role in DNA replication. *Biochim Biophys Acta* **1398**: 113-136.

Lin, Z., Kong, H., Nei, M., and Ma, H. 2006. Origins and evolution of the *recA/RAD51* gene family: evidence for ancient gene duplication and endosymbiotic gene transfer. *Proc Natl Acad Sci* **103**: 10328-10333.

Lin, Z., Nei, M., and Ma, H. 2007. The origins and early evolution of DNA mismatch repair genes--multiple horizontal gene transfers and co-evolution. *Nucleic Acids Res* **35**: 7591-7603.

Malik, S.B., Ramesh, M.A., Hulstrand, A.M., and Logsdon, J.M., Jr. 2007. Protist homologs of the meiotic Spo11 gene and topoisomerase VI reveal an evolutionary history of gene duplication and lineage-specific loss. *Mol Biol Evol* **24**: 2827-2841.

Miki, H., Okada, Y., and Hirokawa, N. 2005. Analysis of the kinesin superfamily: insights into structure and function. *Trends Cell Biol* **15**: 467-476.

Surcel, A., Zhou, X., Quan, L., and Ma, H. 2008. Long-term maintenance of stable copy number in the eukaryotic *SMC* family: origin of a vertebrate meiotic *SMC1* and fate of recent segmental duplicates. *J Syst Evol* **46**: 19.

Zhou, X. and Ma, H. 2008. Evolutionary history of histone demethylase families: distinct evolutionary patterns suggest functional divergence. *BMC Evol Biol* **8**: 294.

Zong, J., Yao, X., Yin, J., Zhang, D., and Ma, H. 2009. Evolution of the RNA-dependent RNA polymerase (RdRP) genes: duplications and possible losses before and after the divergence of major eukaryotic groups. *Gene* **447**: 29-39.

**Table S4: Test of the impact of long-branch attraction on orthogroups with vulnerable topologies.**

|  | Cluster ID | Original support | Support after adding sequences |
| --- | --- | --- | --- |
| (PAF)(P) | 371 | >= 70 | >= 50 |
|  | 287 | >= 50 | Undetermined* |
|  | 478 | >= 50 | >= 50 |
| (PA)(P) | 87 | >= 50 | >= 50 |
|  | 146 | >= 70 | >= 70 |
|  | 696 | >= 50 | >= 50 |
| (PF)(P) | 119 | >= 70 | >= 70 |
|  | 177 | >= 50 | >= 50 |
|  | 716 | >= 70 | >= 50 |
| (PAF)(A) | 187 | >= 70 | >= 70 |
|  | 288 | >= 50 | Undetermined |
|  | 369 | >= 50 | >= 50 |
| (PA)(A) | 63 | >= 50 | >= 70 |
|  | 365 | >= 50 | >= 70 |
|  | 44 | >= 70 | >= 70 |
| (PAF)(F) | 232 | >= 50 | Undetermined |
|  | 396 | >= 70 | >= 70 |
|  | 485 | >= 50 | Undetermined |
| (PF)(F) | 414 | >= 70 | >= 70 |
|  | 711 | >= 50 | No duplication |
|  | 725 | >= 50 | Undetermined |

P – Plants; A – Animals; F – Fungi.

*: Undetermined means the topology could not be resolved at bootstrap support level of 50.

In this analysis, a subset of the orthogroups that have topologies potentially vulnerable to long-branch attraction (LBA) was arbitrarily selected to test the impact of LBA. Additional sequences from the following species were added to each orthogroup:

(PAF)(P), (PA)(P), (PF)(P): *Selaginella moellendorfii* (spikemoss), *Oryza sativa* (rice) and *Vitis vinifera* (winegrape);

(PAF)(A), (PA)(A): *Nematostella vectensis* (sea anemone), *Ciona intestinails* (sea squirt) and *Xenopus tropicalis* (frog);

(PAF)(F), (PF)(F): *Ustilago maydis*, *Aspergillus* and *Neurospora crassa*.

**Table S5:** Distribution of orthogroups with phyletic patterns supporting early eukaryotic duplication – Analysis I

|  | | NJ-BS | | ML-BS | | ML-aLRT | |
| --- | --- | --- | --- | --- | --- | --- | --- |
|  |  | >= 50% | >= 70% | >= 50% | >= 70% | >= 50% | >= 70% |
| (RO)(RO) | (PAF)(PAF) | 46 | 38 | 49 | 33 | 65 | 63 |
|  | (PAF)(PA) | 12 | 6 | 8 | 6 | 12 | 12 |
|  | (PAF)(PF) | 2 | 2 | 3 | 2 | 6 | 5 |
|  | (PA)(PA) | 8 | 5 | 7 | 3 | 11 | 9 |
|  | (PF)(PF) | 1 | 0 | 1 | 0 | 1 | 1 |
|  | (PA)(PF) | 4 | 1 | 3 | 2 | 7 | 5 |
|  | Total | 73 | 52 | 71 | 46 | 102 | 95 |
| (RO)(R) | (PAF)(P) | 25 | 14 | 23 | 13 | 36 | 29 |
|  | (PA)(P) | 25 | 15 | 20 | 10 | 26 | 22 |
|  | (PF)(P) | 6 | 2 | 12 | 6 | 13 | 12 |
|  | Total | 56 | 31 | 55 | 29 | 75 | 63 |
| (RO)(O) | (PAF)(AF) | 1 | 1 | 2 | 1 | 4 | 3 |
|  | (PAF)(A) | 15 | 12 | 16 | 4 | 22 | 21 |
|  | (PAF)(F) | 11 | 6 | 8 | 3 | 9 | 9 |
|  | (PA)(AF) | 0 | 0 | 1 | 1 | 2 | 2 |
|  | (PA)(A) | 25 | 11 | 12 | 7 | 20 | 21 |
|  | (PF)(AF) | 2 | 2 | 2 | 2 | 2 | 2 |
|  | (PF)(F) | 5 | 2 | 5 | 3 | 5 | 4 |
|  | Total | 59 | 34 | 46 | 21 | 64 | 62 |

R – Archaeplastida; O – Opishtokonta; P – Plants; A – Animals; F – Fungi.

**Table S6:** Distribution of orthogroups with phyletic patterns supporting early eukaryotic duplication – Analysis III

|  | | NJ-BS | | ML-BS | | ML-aLRT | |
| --- | --- | --- | --- | --- | --- | --- | --- |
|  |  | >= 50% | >= 70% | >= 50% | >= 70% | >= 50% | >= 70% |
| (RO)(RO) | (PAF)(PAF) | 48 | 23 | 52 | 20 | 158 | 145 |
|  | (PAF)(PA) | 18 | 5 | 18 | 7 | 69 | 59 |
|  | (PAF)(PF) | 4 | 2 | 4 | 2 | 10 | 7 |
|  | (PA)(PA) | 12 | 7 | 13 | 7 | 43 | 40 |
|  | (PF)(PF) | 1 | 0 | 1 | 1 | 1 | 2 |
|  | (PA)(PF) | 7 | 3 | 4 | 2 | 18 | 15 |
|  | Total | 90 | 40 | 92 | 39 | 299 | 268 |
| (RO)(R) | (PAF)(P) | 26 | 15 | 30 | 13 | 67 | 53 |
|  | (PA)(P) | 34 | 16 | 30 | 11 | 58 | 51 |
|  | (PF)(P) | 12 | 10 | 20 | 9 | 31 | 32 |
|  | Total | 72 | 41 | 80 | 33 | 156 | 136 |
| (RO)(O) | (PAF)(AF) | 3 | 1 | 4 | 1 | 8 | 8 |
|  | (PAF)(A) | 38 | 19 | 20 | 7 | 52 | 53 |
|  | (PAF)(F) | 20 | 6 | 10 | 3 | 24 | 28 |
|  | (PA)(AF) | 1 | 0 | 0 | 0 | 7 | 6 |
|  | (PA)(A) | 23 | 11 | 16 | 5 | 54 | 45 |
|  | (PF)(AF) | 0 | 0 | 2 | 1 | 2 | 3 |
|  | (PF)(F) | 9 | 4 | 10 | 5 | 9 | 7 |
|  | Total | 94 | 41 | 62 | 22 | 156 | 150 |

R – Archaeplastida; O – Opishtokonta; P – Plants; A – Animals; F – Fungi.

Table S7: Results of MCL clustering analyses with genes from additional animal species.

|  | Number of clusters with genes from Archeaplastida, Opisthokonta and prokaryotes | Number of eukaryote-specific clusters with genes from both Archeaplastida and Opisthokonta |
| --- | --- | --- |
| Original dataset | 794 | 2276 |
| Adding genes from zebrafish | 804 | 2273 |
| Adding genes from medaka | 807 | 2261 |
| Adding genes from *Drosophila* | 807 | 2271 |
| Adding genes from *Lottia* | 814 | 2336 |
